# Novel keratinolytic enzymes, discovered from a talented and efficient bacterial keratin degrader

**DOI:** 10.1038/s41598-020-66792-2

**Published:** 2020-06-22

**Authors:** Yuhong Huang, Mateusz Łężyk, Florian-Alexander Herbst, Peter Kamp Busk, Lene Lange

**Affiliations:** 1 Department of Biotechnology and Biomedicine, Technical University of Denmark, Building 224, Søltofts Plads, 2800 Kongens Lyngby Denmark; 2 Center for Microbial Communities, Department of Chemistry and Bioscience, Aalborg University, Fredrik Bajers Vej 7H, 9220 Aalborg East, Denmark; 3 Department of Science and Environment, Roskilde University, Universitetsvej 1, 4000 Roskilde, Denmark; 4 Present Address: Beijing Key Laboratory of Ionic Liquids Clean Process, Key Laboratory of Green Process and Engineering, State Key Laboratory of Multiphase Complex Systems, Institute of Process Engineering, Chinese Academy of Sciences, Beijing, 100190 P. R. China; 5 Present Address: Water Supply and Bioeconomy Division, Faculty of Environmental Engineering and Energy, Poznan University of Technology, Berdychowo 4, 60-965 Poznan, Poland; 6 Present Address: Bioeconomy, Research & Advisory, Karensgade 5, DK-2500 Valby, Denmark

**Keywords:** Biological techniques, Biotechnology, Computational biology and bioinformatics, Microbiology, Molecular biology

## Abstract

Huge quantities of keratinaceous waste are a substantial and almost totally unexploited protein resource which could be upgraded for use as high value-added products by efficient keratinolytic enzymes. In this study, we found that *Bacillus* sp. 8A6 can efficiently degrade chicken feather after 24 h growth. According to phylogenetic analysis, the strain (formerly identified as *Bacillus pumilus* 8A6) belongs to the *B. pumilus* species clade but it is more closely related to *B. safensis*. Hotpep predicted 233 putative proteases from *Bacillus* sp. 8A6 genome. Proteomic analysis of culture broths from *Bacillus* sp. 8A6 cultured on chicken feathers or on a mixture of bristles and hooves showed high abundance of proteins with functions related to peptidase activity. Five proteases (one from family M12, one from family S01A, two from family S08A and one from family T3) and four oligopeptide and dipeptide binding proteins were highly expressed when *Bacillus* sp. 8A6 was grown in keratin media compared to LB medium. This study is the first to report that bacterial proteases in families M12, S01A and T3 are involved in keratin degradation together with proteases from family S08.

## Introduction

A very large amount of animal-derived keratinaceous waste such as feathers, skin, hair, bristles, horns, hooves, claws, nails, beaks, reptilian osteoderm, and fish teeth and slime are generated annually^1^. Keratin is the third most abundant polymer in nature after cellulose and chitin^2^. Keratinaceous materials are fibrous proteins composed of recalcitrant polymers with a high degree of cross-linking disulfide bonds, hydrogen bonds, and hydrophobic interactions. The keratin is self-assembled from two polypeptides that form an intermediate filament, sterically mediated by a distinct head and tail structure^1^. Additionally, post-translational modifications, such as the formation of disulfide bonds, phosphorylation and glycosylation leads to various keratin structures and different degrees of recalcitrance and bio-availabilities^3^. The recalcitrant structure of keratin is the first line of defense against microbial attacks on animal body parts.

Molyneux^4^ was the first to isolate a *Bacillus* sp. that was able to degrade keratin. Lin *et al*.^5^ were later the first to purify and characterize keratinase KerA of the S8 protease family from a *B. licheniformis* strain. Subsequently, several different kinds of bacteria with keratinolytic capability have been isolated and described. Single keratinolytic proteases have been purified from culture broth or recombinantly expressed from single strains. However, keratin cannot be degraded by only one keratinase. The complex and recalcitrant structure needs synergistic interaction of different types of keratinolytic enzymes in order to be effectively decomposed^6^.

*Bacillus* species such as *B. licheniformis*, *B. subtilis* and *B. cereus* are well-known for their keratinolytic capability^7–9^. In this study, *Bacillus* sp. 8A6 (deposited at the Bacillus Genetic Stock Center as “*B. pumilus* 8A6”; *B. pumilus* has GRAS status) was found to be an efficient keratin degrader. Based on whole-genome comparisons, the strain studied was found to be more closely related to *B. safensis*. Until now, however, only a few reports have indicated that *B. safensis* has keratin degradation capability and no keratin-degrading enzymes have been identified from this species^10^.

In this study, keratinases from *Bacillus* sp. 8A6 were identified by bioinformatic analysis using Hotpep^11^ and by proteome analysis of culture broths of *Bacillus* sp. 8A6 grown on keratin (chicken feather (CF) or on a mixture of bristles and hooves (BH)). We found five proteases and four oligopeptide and dipeptide binding proteins that were highly up-regulated, i.e. more abundant, when *Bacillus* sp. 8A6 was grown on keratinaceous substrates. These enzymes are likely to be involved in keratin degradation.

## Results

### Strain selection

The *Bacillus pumilus* strain FH9 has exceptional proteolytic enzymes capable of degrading feather waste^12^ and its hydrolytic capacities makes it suitable for use in production of bioplastic^13^. Several other strains of *B. pumilus* are available at the Bacillus Genetic Stock Centre. We chose to use the strain 8A6, which was isolated from a sample taken above the flood line of a stream in a 7 miles long cave in carbonate rock in Kentucky, USA^14^.

### Genome sequencing and *de novo* assembly

The assembly obtained with k-mer value 59 was selected as the best representation of the genome scaffold based on assessment of the constructed deBrujin graphs (highest number of vertices with an ingoing degree of 1 and an outgoing degree of 1) and assembly statistics (highest N_50_ value). Because of the high quality of the reads and coverage of around 400 ×, neither additional trimming nor error correction were necessary and did not improve the assembly when tested. The k-mer value of 59 resulted in 10 contigs with a length >=100 nt; the total length, average length and largest contig length were 3,729,788 bp, 372,987 bp, and 975,857 bp, respectively. The N_50_ was 962,040 bp and 3900 genes were predicted in selected assembly by GeneMarkS. Genome draft completeness was calculated by both BUSCO and CheckM to be 99.6% where 524 out of 526 complete markers and 710 out of 711 complete markers were identified by BUSCO and CheckM, respectively. Duplicated markers were not found, which indicated lack of contamination.

### Classification of *Bacillus* sp. 8A6

**Figure 1:**
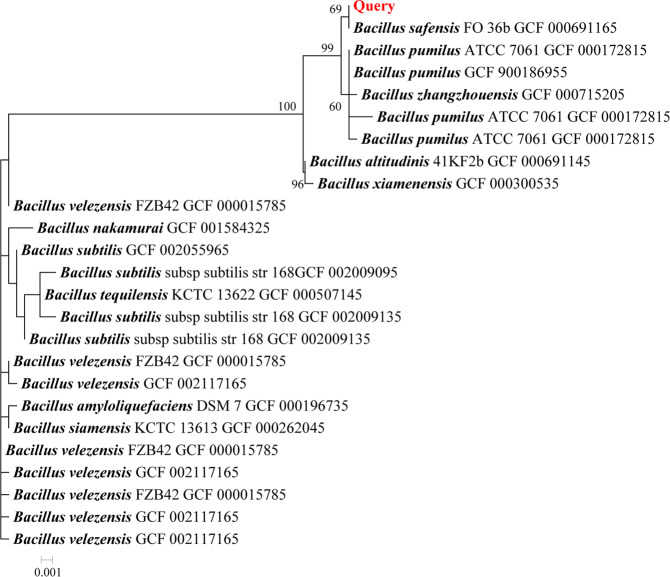
Phylogenetic tree (maximum-likelihood) of the full length 16 S rRNA gene sequences identified in *Bacillus* sp. 8A6 strain (red) and the closest known relatives from type strains. The percentage of replicate trees (>60%) in which the associated taxa clustered together in the bootstrap test (500 replicates) is shown next to the branches.

*Bacillus* sp. 8A6 was first published named only to the level of genus, and then was subsequently deposited in the Bacillus Genetic Stock Center as *Bacillus pumilus* 8A6, based on the 16 S rRNA gene sequence^14^. In this study, the identified 16 S rRNA gene sequence of this strain was compared with 16 S rRNA sequences identified in 202 assemblies of *Bacillus* type strain genomes from the NCBI Assembly database. The most similar sequences identified by BLASTn were subsequently used for phylogenetic analysis (Fig. 1). The 16 S rRNA sequence of *Bacillus* sp. 8A6 formed a distinct clade together with 16 S rRNA sequences from *B. safensis* Fo-36b, *B. pumilus* species and *B. zhangzhouensis* DW5-4. However, species classification of strains related to *B. pumilus* based on the 16 S rRNA gene is not reliable because the majority of them share over 99.5% of 16 S rRNA gene identity^15^. Therefore whole-genome *in silico* comparison using average nucleotide identity (ANI) and Genome-to-Genome distance calculator (GGDC) was applied for more accurate taxonomic placement (Table 1). We found that *Bacillus* sp. 8A6 is more closely related to the *B. safensis* Fo-36b strain than to *B. pumilus* based on significantly higher ANI (98.79%) and digital DNA-DNA hybridization (dDDH) values (89.3), which is higher than the proposed thresholds for species delineation, of 95–96% and 70%, respectively.

**Table 1:** Genome to genome comparisons of the assembled *Bacillus* sp. 8A6 genome and highly related type strains of *Bacillus* species using ANI (OrthoANIu values) and GGDC (DDH values).

Type strain	Genome accession no.	Ortho ANIu algorithm		GGDC			G + C diff.
		OrthoANIu value (%)	8A6 Coverage (%)	dDDH	Distance	Prob. DDH >=70%	
*B. safensis* FO-36b	GCF_000691165.1	98.79	71.91	89.3	0.0129	95.54	0.02
*B. pumilus* ATCC 7061	GCF_000172815.1	91.77	62.65	44.7	0.0851	7.74	0.04
*B. zhangzhouensis* DW5-4	GCF_000715205.1	90.86	62.72	42.2	0.0925	4.53	0.25
*B. xiamensis* HYC-10	GCF_000300535.1	89.29	59.46	37.4	0.1087	1.35	0.33
*B. altitudinis* 41KF2b	GCF_000691145.1	88.94	64.21	36.3	0.1128	0.99	0.36

### Proteases prediction by Hotpep analysis in *Bacillus* sp. 8A6 and *B. safensis* Fo-36b genomes

**Figure 2:**
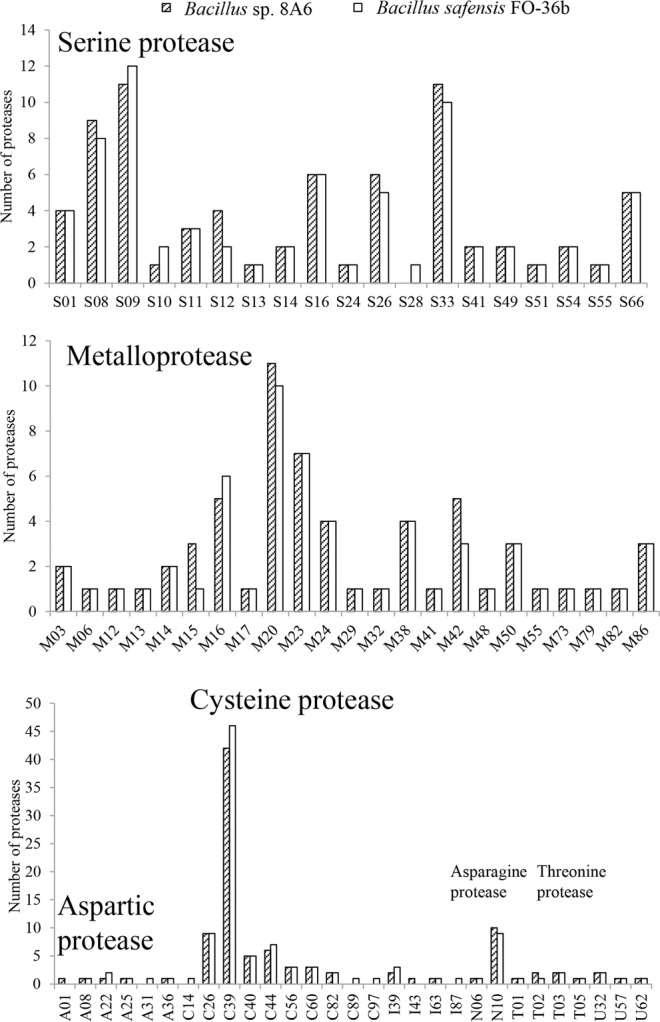
Protease profiles (from genome annotation) of *Bacillus* sp. 8A6 and *B. safensis* Fo-36b.

Proteases were predicted in both *Bacillus* sp. 8A6 and *B. safensis* Fo-36b genomes by Hotpep analysis. All the predicted sequences were supported by CDD search and blasting in the Merops database. Finally, 233 and 235 proteases in Aspartic (A), Cysteine (C), Metallo (M), Asparagine (N), Serine (S), Threonine (T) and Unknown (U) protease/peptidase families were found in *Bacillus* sp. 8A6 and *B. safensis* Fo-36b genomes, respectively (Fig. 2). The protease profiles of both strains were similar, especially for serine proteases and metalloproteases. Potential keratinolytic proteases of *Bacillus* sp. 8A6, *B. safensis* Fo-36b, *B. pumilus* SH-B9, *B. altitudinis* 41KF2b, *B. stratosphericus* LAMA585 and *B. safensis* KCTC12796BP were found in 10 protease families (A1, A8, A22, A24A, A25, A28, A31, A36, M03B and S08A) according to functional prediction of keratinolytic proteases from fungi and bacteria (Supplementary Table S1). 16 potential keratinolytic protease sequences were found in the *Bacillus* sp. 8A6 genome.

### Proteomic analysis of keratinolytic protease genes in *Bacillus* sp. 8A6

#### Efficient degradation of keratin by Bacillus sp. 8A6

*Bacillus* sp. 8A6 showed high capability for keratin degradation when grown in liquid culture including keratinaceous substrate. This strain efficiently degraded CF in 24 h at 37 °C when using CF as sole carbon and nitrogen source. Keratinase activity when using CF or BH as substrate was 4,463 and 4,043 U/ml, respectively, while keratinase activity was only 790 U/ml when grown in LB medium. Moreover, protease activity when *Bacillus* sp. 8A6 was grown in medium with CF or with BH (33,650 and 30,500 U/ml, respectively) was higher than when grown in LB medium (3,917 U/ml). After keratin degradation by *Bacillus* sp. 8A6, up to 3.79 and 9.55 mg/ml soluble protein were released into the medium from CF and BH, respectively.

#### MS analysis of protein composition of secretome of Bacillus sp. 8A6

**Figure 3:**
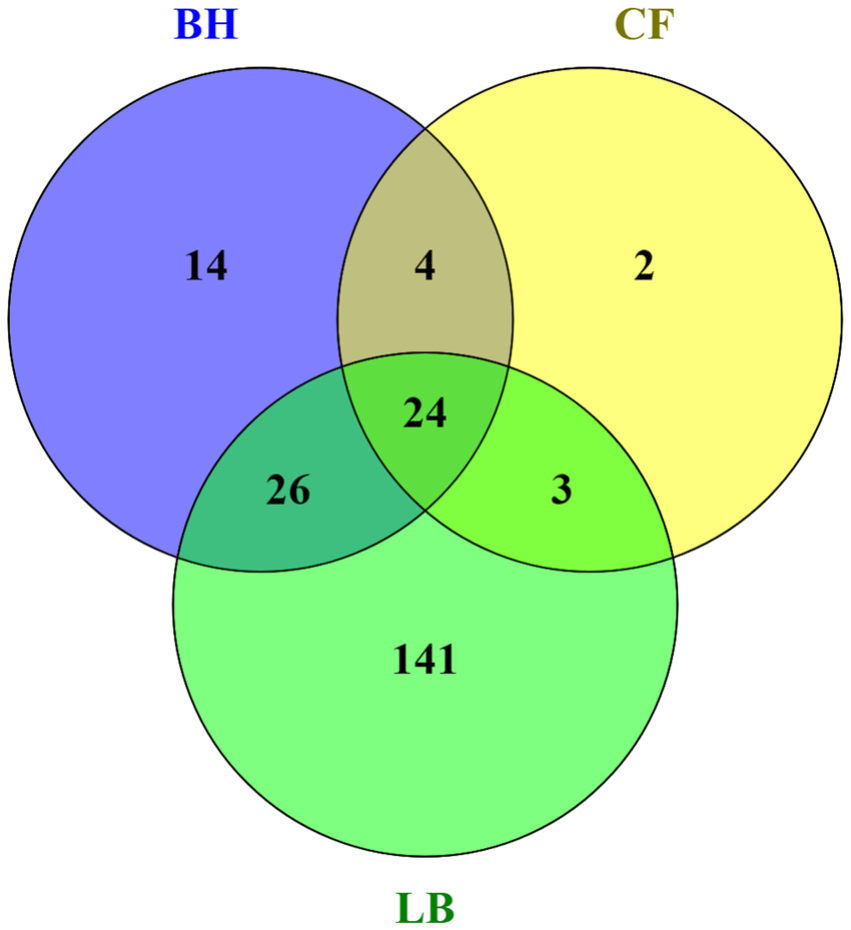
Venn diagram of all the identified proteins in the secretome of *Bacillus* sp. 8A6 grown in chicken feather (CF), bristles and hooves (BH) and LB medium.

**Figure 4:**
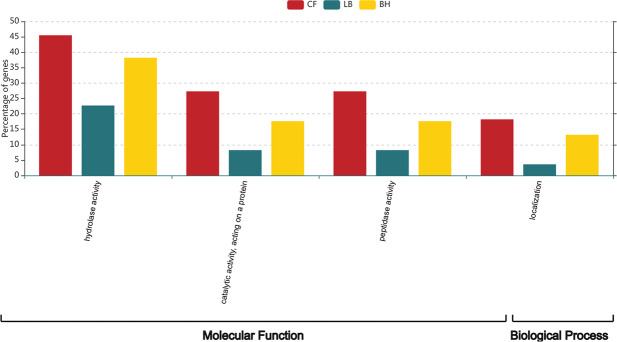
Percentage of genes with ontologies identified as differentially abundant in secretome of *Bacillus* sp. 8A6 grown on chicken feather (CF), bristles and hooves (BH) and LB medium.

In total, 194, 68, and 33 proteins were identified in culture broth from *Bacillus* sp. 8A6 grown in LB, BH and CF medium, respectively (Fig. 3). According to the GO ontology comparison of proteins detected in secretome samples (Supplementary Fig. S1), proteins with functions related to peptidase activity, catalytic activity acting on a protein and hydrolase activity in molecular functions showed significantly higher abundance when *Bacillus* sp. 8A6 was grown in the keratin media than in LB medium (Fig. 4 and Supplementary Fig. S2).

Of the proteins with annotated function, four proteins (derived from gene_2558: GH16_lichenase, gene_2946: xylanase, gene_3650: the substrate-binding component of the oligopeptide-binding protein, and gene_9: the substrate-binding component of an ABC-type dipeptide import system containing the type 2 periplasmic binding fold) were found only in culture broth with BH and CF media. According to the t-test results for culture broth from keratin media against the results for culture broth of LB medium, six proteases (one from family M12, two from family S08A, one from family S01A, one from family T3 and one from family M17) showed statistically significant up-regulation according to the –LOG (P-value) when *Bacillus* sp. 8A6 was grown on CF (Table 2, Supplementary Table S2). Furthermore, seven proteases (one from family M12, one from family S01A, three from family S08A, one from family M20B and one in family T3) showed statistically significant up-regulation when *Bacillus* sp. 8A6 was grown on BH medium (Table 3, Supplementary Table S3). Thus, when *Bacillus* sp. 8A6 was grown on keratinaceous substrates, five proteases (derived from gene_1796 in family M12, gene_3018 in family S01A, gene_3289 and gene_3746 in family S08A, and gene_3552 in family T3) were shown to be highly up-regulated.

**Table 2:** Up-regulated proteases in the secretome of *Bacillus* sp. 8A6 grown in chicken feather (CF) medium compared to in LB medium. Detailed information can be found in Supplementary Table S2.

Gene ID	Merops family	Annotation	Function	Signal peptide	Accession number
gene_1796	M12	Metallo-peptidase family	metalloendopeptidase	+	MH476086
gene_3289	S08A	Peptidases_S8_Bacillopeptidase	serine endopeptidase	+	MH476112
gene_3018	S01A	Trypsin-like serine protease	serine endopeptidase	+	MH476104
gene_3746	S08A	Peptidases_S8_Subtilisin	serine endopeptidase	+	MH476105
gene_3552	T3	Gamma-glutamyltranspeptidase	aminopeptidase aminotransferase	+	MH476180
gene_2289	M17	Multifunctional aminopeptidase A	aminopeptidases	−	MH476039

**Table 3:** Up-regulated proteases in the secretome of *Bacillus* sp. 8A6 grown on bristles and hooves (BH) compared to LB medium. Detailed information can be found in Supplementary Table S3.

Gene ID	Merops family	Annotation	Function	Signal peptide	Accession number
gene_1796	M12	Metallo-peptidase	metalloendopeptidases	+	MH476086
gene_3018	S01A	Trypsin-like serine protease	serine endopeptidases	+	MH476104
gene_3746	S08A	Peptidases_S8_Subtilisin	serine endopeptidase	+	MH476105
gene_3289	S08A	Peptidases_S8_Bacillopeptidase	serine endopeptidase	+	MH476112
gene_1734	S08A	Peptidases_S8_subtilisin	serine endopeptidase	+	MH476109
gene_1093	M20B	M20_peptT_like	exopeptidases	−	MH476041
gene_3552	T3	Gamma-glutamyltranspeptidase	aminopeptidase aminotransferase	+	MH476180

In this study we also found that the substrate binding component for oligopeptide and dipeptide, such as gene_3645, was highly up-regulated in the culture broth with CF and BH (Table 4). Furthermore, three other substrate binding components (gene_3650, gene_9 and gene_992) could also be found only in the keratin medium but not in the LB medium (Table 4).

**Table 4:** Up-regulated substrate-binding components in the secretome of *Bacillus* sp. 8A6 grown on chicken feather (CF) and on bristles and hooves (BH) compared to LB medium. Detailed information can be found in Supplementary Table S2 and Table S3.

	Gene ID	Function	Accession number
Highly up-regulated in keratin medium	gene_3645	The substrate-binding component of an ABC-type oligopetide import system contains the type 2 periplasmic binding fold	MH476185
Only detected in keratin medium	gene_3650	The substrate-binding component of the oligopeptide-binding protein	MH476186
	gene_9	The substrate-binding component of an ABC-type dipeptide import system	MH476187
	gene_992	Substrate binding domain of putative ABC-type phosphate transporter	MH476188

## Discussion

Keratinaceous waste streams such as feathers, pig bristles and hooves have high amounts of intermediate filament-forming proteins with specific physicochemical properties. Recently, upgrade not just of lignocellulose and chitin but also of keratinaceous waste has been studied and discussed in relation to the new bioeconomy^2,16^. Several types of bacteria and fungi have been found to process sets of enzymes needed for keratinolytic capability^2^. Specialized fungi such as saprotrophs growing on e.g. feather and hooves (as e.g. *Onygena corvina*)^17^ or dermatophytic species, have been shown to have a rich diversity of keratinolytic enzymes^18^. However, the fungal enzymatic keratinolytic process appears to be rather slow, taking a longer time (e.g.> 10 days) than is easily accommodated in an industrial setting of waste conversion^17^. Interestingly, and in contrast, some bacteria have optimum keratinase activity production at around 30–72 h. These species include *B. licheniformis* PWD-1^9^, *B. licheniformis* RG1^19^, *Fervidobacterium pennavorans*^20^, *Fervidobacterium islandicum* AW-1^21^ and *Meiothermus taiwanensis* WR-2230^22^ which can completely degrade keratin in a short time (24–48 h), excepting *B. licheniformis* PWD-1 which needs 10 days. However, these species are slower than the *Bacillus* sp. 8A6 studied here regarding keratin degradation. In this study, we found that *Bacillus* sp. 8A6 efficiently degraded CF and that the keratinase activity of the culture broth was at its highest within only 24 h of incubation. This strain was found also to produce the highest keratinase activity after only 18 h growth in a 5 L fermenter when using BH as substrate (data not shown). These findings are a motivation for further characterization of the highly efficient keratinolytic proteases from *Bacillus* sp. 8A6.

In this study we investigated the taxonomy of *Bacillus* sp. 8A6 by supplementing the 16 S rRNA phylogenetic approach with whole-genome *in silico* comparisons. The designation of the species within the “*B. pumilus*” group based solely on ribosomal gene markers is not reliable because most of the group members share high 16 S rRNA gene identity. For example, Espariz *et al*.^15^ found that as many as 50% of strains in this group were misclassified. This highlights the necessity of utilizing more detailed phylogenomic and whole-genome based approaches for more specific classification of organisms in the *B. pumilus* complex. Based on comparison of the *Bacillus* sp. 8A6 genome reported in this study with available genomes of type strains belonging to the “*B. pumilus* species group”, we propose that strain *B. pumilus* 8A6 be renamed as *B. safensis* 8A6. *B. safensis* was first identified in 2006 as a contaminant in the spacecraft assembly facility at the Jet Propulsion Laboratory, USA, from which it derived its specific epithet ‘safensis’^23^. Although *B. safensis* does not have GRAS status yet, it has been regarded as a safe industrial microorganism with promising biotechnological applications due to its ability to produce various industrial enzymes and secondary metabolites^24^. Until now, only *B. safensis* LAU 13 has been reported to have keratinolytic activity^10,25^. However, specific keratinolytic proteases have not been identified nor characterized from *B. safensis*.

The Hotpep predicted protease profile of *Bacillus* sp. 8A6 was similar to *B. safensis* FO-36b. We also attempted to investigate whether or not *Bacillus* sp. 8A6 and other *Bacillus* species have lytic polysaccharide monooxygenases (LPMOs). Notably, LPMO AA11 is widely found in keratin degrading fungi^26^. Further, AA11 was described from the keratin degrading fungus, *O. corvina*. Based on detailed study of the keratinolytic capability of *O. corvina*, it was hypothesized that LPMO AA11 is important for the keratinolytic effect of this fungus^2^. In contrast, no LPMO protein could be found by HotPep analysis in *Bacillus* sp. 8A6. Similarly, LPMOs were also not found in the genome of *B. safensis* Fo-36b, *B. pumilus* SH-B9, *B. aerophilus* C772, *B. altitudinis* 41KF2b, *B. stratosphericus* LAMA585 or *B. safensis* KCTC12796BP. The apparent lack of LPMO genes in the genome of keratinolytic bacteria suggests that bacteria may have a different mechanism(s) for keratin degradation than fungi. Notably, the predicted potential keratinolytic proteases belonging to protease families A1, A8, A22, A24A, A25, A28, A31, A36, M03B and S08A in the above bacterial species are also different from those of fungi such as *O. corvina*, which mainly has proteases with keratinolytic activity from families M03, M28 and S8^6^.

The first keratinase described from *B. licheniformis* strain were serine protease in family S8^5^. Recently, a broader diversity of keratinolytic bacteria has been found, but most of the identified bacterial keratinolytic proteases were actually in family S8^27^. All the commercial keratinases were classified in the S8 family^2^. However, not all the proteases in family S8 have high keratinolytic capability. In this study, the two proteases from family S8 showed high up-regulation when *Bacillus* sp. 8A6 was grown on CF or BH. Some other proteases in family S8 did not show any significant difference when the secretome of *Bacillus* sp. 8A6 grown in keratin medium and in LB medium was compared.

Only few proteases of M12 family have been found from bacteria since 1995^28^. The M12 metalloproteases from the deep-sea bacterium *Myroides profundi* D25 was found to have elastinolytic activity and a synergistic role in collagen hydrolysis^28^. This specific swollen collagen structure property was further confirmed by an optimized scale up process^29^. The most highly up-regulated M12 metalloprotease of *Bacillus* sp. 8A6 when grown in keratin medium might indicate that this metalloprotease M12 can modify the structure of the keratin, which as a result then becomes accessible for other keratinolytic proteases.

In this study, a non-keratin-specific trypsin-like protease from family S01A was also found to be abundant in the studied keratinolytic culture broths. In 2001, a new trypsin-like protease was first isolated from a *B. licheniformis* strain when grown in fermentation broth with milled chicken feather^30^. However, the function of this trypsin-like protease during keratin degradation has never been clarified. *Streptomycetes exfoliatus* and *S. albidoflavus* were found to produce trypsin when grown under limiting carbon, nitrogen and phosphate growth conditions^31^. The production of trypsin by *S. exfoliatus* and *S. albidoflavus* was also positively related to aerial Streptomycete-mycelium formation and growth^32^. Therefore, the up-regulated trypsin-like protease in keratin-rich medium might be induced by insoluble CF and BH and may further degrade oligopeptides produced by specific keratinolytic proteases. It is also found that multifunctional aminopeptidase A in M17 family has been highly up-regulated when *Bacillus* sp. 8A6 grown on chicken feather, which might participate for further degrading oligopeptides as the function of the aminopeptidase from *O. corvina*^6^.

Feathers, bristles and hooves are cysteine-rich keratin-associated recalcitrant protein structures^33^. After the feathers, bristles and hooves have been degraded by the keratinolytic proteases, the resulting oligopeptides will also be rich in cysteine, which opens the way for production of e.g. glutathione. The secreted gamma-glutamyl transpeptidase in family T3 from *Bacillus* sp. 8A6 can hydrolyze glutathione to glutaminate and cysteinyl-glycine, which can be further hydrolyzed to cysteine and glycine. The resulting glutaminate, cysteine and glycine can be taken up by the microbial cell directly. It has been reported that gamma-glutamyl transpeptidase can balance the levels of intracellular cysteine^30,34^. Grumbt *et al*.^35^ have proposed that during keratin degradation by dermatophytes, intracellular cysteine can be metabolized to sulfite via the action of the enzyme cysteine dioxygenase Cdo1. Then the released sulfite further facilitates keratin degradation. Therefore, the up-regulated gamma-glutamyl transpeptidase in the keratin medium not only provides the cell with cysteine but also with, the further metabolized products that may also support the keratin degradation process itself. Disulfide reductases are also well-known for participating the keratin degradation^36^; however, we did not find disulfide reductases in the secretome when *Bacillus* sp. 8A6 was grown on CF or BH.

Besides the keratinolytic proteases, the mass spectrometric analyses also identified four oligopeptide and dipeptide substrate binding proteins. These substrate binding proteins were the membrane-associated components of an ATP-binding cassette (ABC) transport system OppABCDEF consisting of five subunits. The substrate-binding components determined the substrate specificity of the transport system. It has been found that this ABC transport system has an important role in supplying bacteria with essential amino acids in a low energy-requiring process^37^. This system appears to be very important for the keratinolytic microorganism’s uptake of oligopeptides or amino acid from the medium after the keratin degradation^18^.

On the basis of the comprehensive genome and secretome analysis, we therefore propose the synthetic effects of different enzymes for keratin degradation by *Bacillus* sp. 8A6: The structure of hard keratin is first weakened by metabolized product sulfite and M12 metalloprotease, and subsequently the loosened structure of keratin is further hydrolyzed by S08A serine proteases. The resulting long peptides are further degraded by a S01A serine protease and the oligopeptides and dipeptides are transported into the cell by an ABC transport system. The secreted family T3 gamma-glutamyl transpeptidase provides the cell with cysteine while additional metabolized products such as sulfite might also support the keratin degradation process. Detailed characterization of each enzymes is part of ongoing research.

In conclusion, this study showed that *Bacillus* sp. 8A6 strain could efficiently degrade CF and BH, paving the way for further improvements in utilization of keratin-rich materials. Phylogenetic analysis revealed that formerly identified as *B. pumilu*s, strain 8A6 is more properly classified as *B. safensis*. Hotpep analysis predicted 233 proteases and 16 potential keratinolytic proteases in the *Bacillus* sp. 8A6 genome. Proteomic analysis of *Bacillus* sp. 8A6 secretome showed a significant induction of proteins related to peptidase activity by keratin. Five proteases (one from family M12, one from family S01A, two from family S8A and one from family T3) and four oligopeptide and dipeptide binding components were highly expressed both in CF and in BH medium compared to LB medium. This study is the first to suggest the involvement of proteases of families M12, S01A, T3 and S8 in keratin degradation.

## Methods

### Microorganism and growth conditions

*Bacillus* sp. 8A6 (formerly identified as *Bacillus pumilus* 8A6) (wild type isolate, original code GGC-D3, BGSCID 8A6) was obtained from the Bacillus Genetic Stock Center. This strain was grown on LB agar medium. For keratinolytic enzymes production, a single clone of *Bacillus* sp. 8A6 was inoculated in 100 ml LB medium and incubated at 37 °C for 20 h (OD600: 1.7–2). Harvest of cells was performed by centrifugation at 6000 g for 5 min and the cell pellet was resuspended in 50 ml PBS buffer (pH 7.5). Two ml of the resuspended cells was inoculated into 50 ml mineral keratin medium (3% BH/1% CF, 0.75 g/l NaCl, 1.75 g/l K_2_HPO_4_, 0.25 g/l MgSO_4_·7H_2_O, 0.055 g/l CaCl_2_, 0.010 g/l FeSO_4_·7H_2_O, 0.005 g/l ZnSO_4_·7 H_2_O, 10 mM MOPS, pH 8) or into 50 ml LB medium as negative control. The culture and control were incubated at 37 °C on a rotary shaker (180 rpm) for 24 h.

The pig bristles and hooves mixture (BH), which have been chopped, steam treated (150 °C, 6 bar, 20 min), dried and crushed into smaller particles, were supplied by DAKA, Saria group, Løsning, Denmark. Chicken feathers (CF) were obtained from Rose Poultry (Vinderup, Skovsgaard, Denmark). The concentration of BH and CF in the medium was determined according to Huang *et al*., manuscript in preparation.

### Enzyme analysis and protein determination

Protease activity was analyzed as described^6^ by using azocasein as substrate (Megazyme) with a small modification. First, 20 μl 1.5% w/v azocasein suspension was mixed with 50 mM sodium carbonate buffer pH9 and 20 μl diluted enzyme in 1.5 ml tubes. The reactions were then conducted at 60 °C for 15 min with constant agitation at 500 rpm (Huang *et al*., manuscript in preparation). One arbitrary unit (U) of protease activity was defined as the amount of enzyme causing a 0.01 absorbance increase between the sample and control at 405 nm under the assay conditions.

Keratinolytic protease activity was analyzed using azokeratin (kindly provided by Professor Søren Sørensen’s group, Section of Microbiology, University of Copenhagen). 500 µl diluted enzyme was added to 800 µl of 0.01 g/ml azokeratin in 50 mM sodium carbonate buffer pH9. The reaction was carried out at 60 °C and 1000 rpm for 1 h (Huang *et al*., manuscript in preparation). Then the mixture was centrifuged at 16,000 g for 1 min and 150 μl supernatant was transferred to a microtiter plate. Absorbance was read at 415 nm using a plate reader. A control was prepared using 500 µl 50 mM sodium carbonate buffer pH9 to replace the addition of 500 µl diluted enzyme. One arbitrary unit (U) of keratinolytic protease activity was defined as the amount of enzyme causing a 0.001 absorbance increase between the sample and control at 415 nm under the assay conditions.

Culture broth protein concentration was determined by PIERCE BCA Protein Assay Kit (23225, Thermo Scientific) using BSA as standards.

### Genomic DNA extraction and sequencing

*Bacillus* sp. 8A6 was grown in LB medium for 20 h at 37 °C and 180 rpm and then 1 ml of culture was centrifuged at 5000 g for 10 min. The genomic DNA was extracted using DNeasy Blood & Tissue Kit (Qiagen, Cat No./ID: 69506) according to a protocol for gram-positive bacteria. The quantity and quality of the genomic DNA was checked on a NanoDrop 1000 spectrophotometer (THERMO ECIENTIFIC) and by electrophoresis on 1% agarose gel. The sample was submitted to Macrogen (Korea) for Truseq PCR-free (350 bp) shotgun library preparation and sequencing using an Illumina HiSeq. 2500 instrument (150 bp paired-end sequencing).

### *De novo* genome assembly and open-reading frame prediction

The last bases (101) were removed from all reads. The read-through adapters on the 3’ ends were trimmed by using k-mer based detection implemented in BBDuk (settings: hdist = 1, k = 21, mink = 11, tbo, tpe)^38^. Reads were quality-trimmed using a Phred algorithm (to a value higher than 20). Assembly was performed using Ray (de Bruijin graph-based assembly) with varying k-mer length (35, 39, 43, 49, 55, 59, 65, 75, 95)^39^. The constructed library had an average outer distance between reads (insert length of the library) of 385 ± 83. Open reading frames were predicted with GeneMarkS^40^ with combined GeneMarkS generated (native) and Heuristic model parameters integrated into one model. The genome draft completeness was assessed by BUSCO (v3), with lineage-specific ortholog set for *Bacillales* (*odb9*), and CheckM v1.0.9 with lineage-specific marker set for *Bacillus* genus (UID864). Both approaches use collocated sets of genes that are ubiquitous and single-copy within a phylogenetic lineage^41,42^.

### Protease prediction by Hotpep analysis

The protease sequences were first predicted by Hotpep analysis using the same approach as described for CAZymes^11^. The predicted protease sequences were then further supported by batch CDD searching in NCBI and blasting in the MEROPS database 12.0 (file “pepunit.lib”)^43^ (2018, March) using CLC Main Workbench version 7 with default parameters.

### Phylogenetic analysis of 16 S rRNA

The phylogenetic analysis was performed as follows. Ribosomal 16 S RNA gene sequences were predicted using barrnap 0.7^44^. One full length 16 S rRNA sequence was identified. The reference database was prepared from 16 S RNA gene sequences identified in 202 assemblies of *Bacillus* type strains’ genomes from NCBI Assembly database. The identified 16 S gene was submitted to a BLAST analysis against the reference database and 50 hits (>1,000 bp, *E* value <1e-20) with the highest identities (>95%) were extracted. Reference sequences were de-replicated and together with query sequence aligned to the RF00177 model from the Rfam database using SSU- ALIGN^45^. SSU-mask was applied for automated probabilistic masking. A maximum-likelihood (ML) phylogenetic tree was constructed using MEGA 7 with the general time-reversible model with correction for among site rate variation (Gamma distributed with Invariant sites, G + I). A bootstrap replication of 500 was used for testing the phylogeny.

### Genome to genome comparisons

The genomes of five highly related type strains of *Bacillus* species (Table 1) were obtained from the NCBI Assembly database (March 2018). These downloaded genomes and the draft genome of *Bacillus* sp. 8A6 were compared with the GGDC webservice at http://ggdc.dsmz.de (BLAST + as an alignment tool and sum of all identities found in HSPs divided by overall HSP length as a distance measure)^46^ and the ANI Calculator webservice at https://www.ezbiocloud.net/tools/ani^47^.

### Precipitation of proteins in culture broth supernatant of *Bacillus* sp. 8A6 for MS analysis

Culture broth of *Bacillus* sp. 8A6 grown in medium with either CF, BH or LB medium, was harvested by centrifugation at 10000 g for 15 min at 4 °C. The supernatant was filtered (0.2 µm). The extracellular proteins were precipitated as described^6^. Prior to LC-MS/MS measurements, proteins were in-solution digested using trypsin as described before^6^. After tryptic digestion, peptides were purified using C18 packed StageTips^48^ and dried by vacuum centrifugation.

### Analysis of proteins by LC-MS/MS

Peptides were reconstituted in 0.1% trifluoroacetic acid/2% acetonitrile solution. Eight microliters of each sample were injected by autosampler and concentrated as well as washed on a trapping column (Pepmap100, C18, 100 μm × 2 cm, 5 μm, THERMO FISHER SCIENTIFIC) with water containing 0.1% formic acid at 800 bar. Afterwards, the peptides were eluted from a separation column (PepmapRSLC, C18, 75 μm×50 cm, 2 μm, THERMO FISHER SCIENTIFIC). Chromatography was performed with 0.1% formic acid in solvent A (100% water) and B (80% acetonitrile, 20% water). Three successive linear gradients with solvent B were run one after another. First, from 5% to 12% within 5 min, next from 12 to 37% over 50 min, and then from 37 to 50% within 5 min, followed by a final one-minute-step gradient to 100% solvent B, which was maintained for 20 min using a nano-high-pressure liquid chromatography system (Easy-nLC 1200, THERMO FISHER SCIENTIFIC). Ionized peptides were measured and fragmented by a Q Exactive mass spectrometer (THERMO FISHER SCIENTIFIC). For an unbiased analysis, continuous scanning of eluted peptide ions was carried out between 400 and 12,000 m/z, automatically switching to MS/MS higher energy collisional dissociation (HCD) mode and 12 MS/MS events per survey scan. For MS/MS HCD measurements, a dynamic precursor exclusion of 30 s, peptide match, and an apex trigger of 2 to 20 s were enabled.

### MS data analysis

Protein identification was done with the open-source software MaxQuant (v. 1.5.3.30)^49^. The label-free quantification (LFQ) algorithm^50^, IBAQ, and the match between runs feature were activated. Carbamidomethylation of cysteines was defined as fixed modification, and oxidation of methionines as well as *N*-terminal acetylation was defined as variable modification. The remaining settings were kept at default. This included a maximum peptide and protein false discovery rate of 1% and a minimum of two peptides for LFQ calculation. The predicted proteins of *Bacillus* sp. 8A6 database after the GeneMarkS prediction as described above was used as a search database in MaxQuant. The mean LFQ per protein was calculated if a protein was quantified in at least two out of three biological replicates. For comparison of relative changes, the LFQ ratios between conditions were formed and log2 transformed. Statistical significances of abundance changes were assessed by t-test (two-tailed, heteroscedastic). Batch CD search^51^ was used to search for conserved domains and annotation of identified protein. A Venn diagram was created by Venny 2.1^52^. Sequences of proteins identified in secretomes were subjected to annotation with the InterProScan pipeline standalone suite (5.27–66.0) using all possible analyses and databases^53^. GO annotations for each dataset were subsequently compared with WEGO^54^.

## Supplementary information

Supplementary Information.

## Data Availability

The genome sequence of *Bacillus* sp. 8A6 has been submitted to the Genbank database under accession number QFZE00000000. The Hotpep predicted protease nucleotide sequences in *Bacillus* sp. 8A6 genome and the up-regulated protein nucleotide sequences in the secretome when *Bacillus* sp. 8A6 was grown in keratin media compared to LB medium have been submitted to the Genbank database under accession numbers MH475952-MH476188 (Tables 2, 3, 4 and Supplementary Table S4)
