# Supplementary material for: Novel keratinolytic enzymes, discovered from a talented and efficient bacterial keratin degrader
Source: Sci Rep. 2020 Jun 22;10:10033. doi: 10.1038/s41598-020-66792-2 (PMC7308268; doi:10.1038/s41598-020-66792-2)
Supplement: Supplementary file 1 — Supplementary Information. [file 41598_2020_66792_MOESM1_ESM.pdf]

Supplementary Information's for

Novel keratinolytic enzymes, discovered from a talented and efficient bacterial keratin degrader

Yuhong Huang[1,4], Mateusz Łężyk[1,5], Florian-Alexander Herbst[2], Peter Kamp Busk[1,3] , Lene Lange[1,6*]

[1]Department of Biotechnology and Biomedicine, Technical University of Denmark, Building 224, Søltofts Plads, 2800, Kongens Lyngby, Denmark

[2]Center for Microbial Communities, Department of Chemistry and Bioscience, Aalborg University, Fredrik Bajers Vej 7H, 9220 Aalborg East, Denmark

[3]Department of Science and Environment, Roskilde University, Universitetsvej 1, 4000 Roskilde, Denmark

[4]Present address: Beijing Key Laboratory of Ionic Liquids Clean Process, Key Laboratory of Green Process and Engineering, State Key Laboratory of Multiphase Complex Systems, Institute of Process Engineering, Chinese Academy of Sciences, Beijing 100190, P. R. China

[5]Present address: Water Supply and Bioeconomy Division, Faculty of Environmental Engineering and Energy, Poznan University of Technology, Berdychowo 4, 60-965 Poznan, Poland

[6]Present address: Bioeconomy, *Research & Advisory*, Karensgade 5, DK-2500 Valby, Denmark

Correspondence and requests for materials should be addressed to senior author LL (Phone: +45 24432040; Email: lene.lange2@gmail.com)

Supplementary Figures

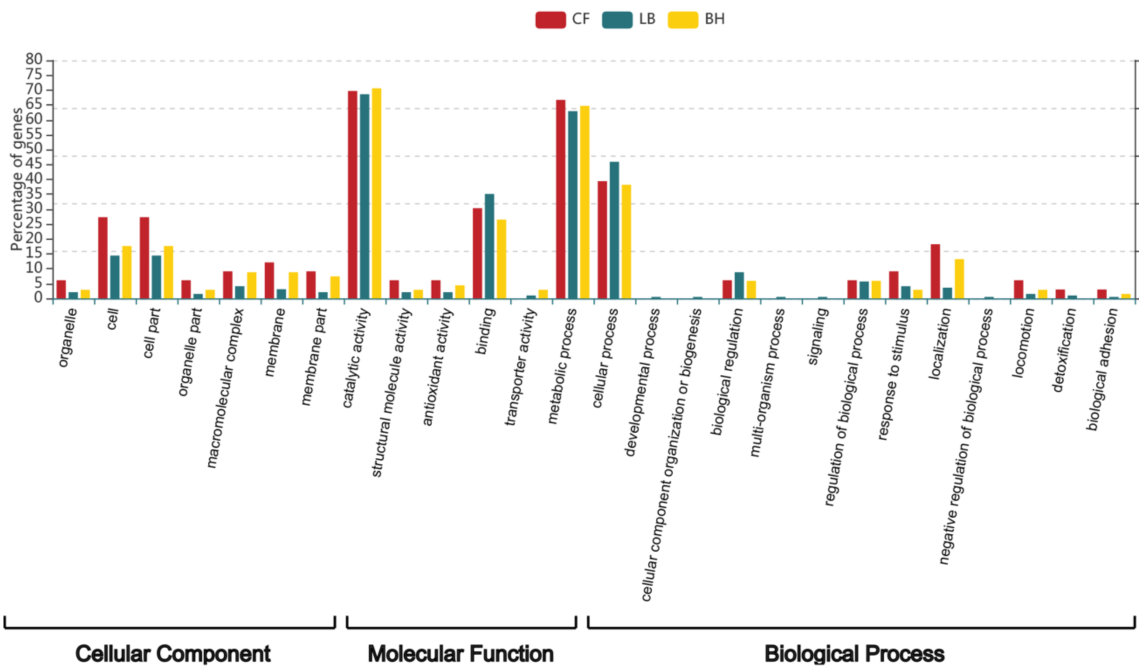

Fig. S1 Functional annotation of identified protein in the secretome of *Bacillus* sp. 8A grown on chicken feather (CF), bristles and hooves (BH) and LB medium

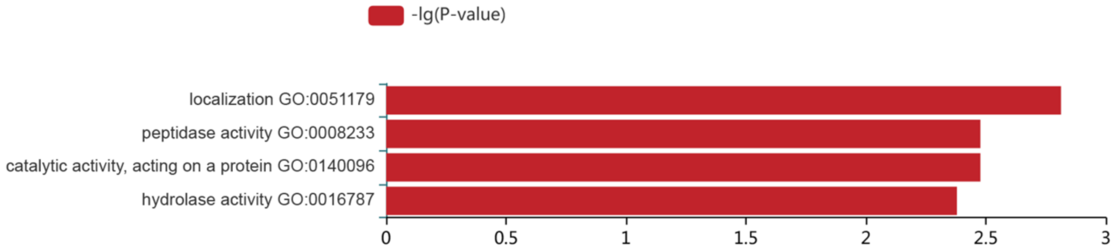

Fig. S2 Statistical significance of differences between number of genes of identified proteins in the secretome of *Bacillus* sp. 8A6 grown on chicken feather (CF), bristles and hooves (BH) and LB medium

Supplementary Tables

Table S1 Potential keratinolytic proteases in *Bacillus* species

Proteases family	*Bacillus* sp. 8A6	*B. safensis* Fo-36b	*B. safensis* KCTC12796BP	*B. pumilus* SH-B9	*B. aerophilus* C772	*B. altitudinis* 41KF2b	*B. stratosphericus* LAMA585
A01	1						
A08	1	1	1	1	1	2	1
A22	1	2					
A24A							1
A25	1	1	1	1	1	1	1
A28			1	1	1	1	1
A31		1					
A36	1	1	1	1	1	1	1
M03B	2	2	2	2	2	2	2
S08A	9	8	8	9	8	9	8

Table S2 T-test results of the proteins in the secretome of *Bacillus* sp. 8A grown on chicken feather (CF) against proteins from *Bacillus* sp. 8A6 grown on LB medium. The LFQ ratios between conditions were formed and log2 transformed to compare the relative changes (difference). Statistical significances of abundance changes were assessed by t-test (two-tailed, heteroscedastic).

Protein IDs	Function	Significant	-LOG(P-value)	Difference	LFQ intensity BH1	LFQ intensity BH2	LFQ intensity BH3	LFQ intensity CF1	LFQ intensity CF2	LFQ intensity CF3	LFQ intensity LB1	LFQ intensity LB2	LFQ intensity LB3
gene_1796	Metallo-peptidase family M12	+	3.308	3.993	24.990	24.573	25.754	26.265	26.282	26.620	23.044	21.770	22.374
gene_3289	Peptidases_S8_BacillopeptidaseF-like	+	2.444	3.017	25.265	25.266	26.308	26.587	26.800	NaN	23.748	23.172	24.110
gene_3018	Trypsin-like serine protease	+	1.780	2.460	27.970	27.793	28.041	27.614	27.418	28.515	25.941	25.876	24.350
gene_3746	Peptidases_S8_Subtilisin_subset	+	1.105	2.327	28.961	28.796	29.231	29.920	29.848	27.079	26.922	25.972	26.973
gene_3857	metallophosphatase superfamily,	+	2.123	1.392	NaN	NaN	NaN	24.523	NaN	24.178	22.956	23.191	22.727
gene_3552	Gamma-glutamyltranspeptidase	+	2.335	1.275	28.181	27.781	28.218	28.794	28.297	28.142	27.070	26.996	27.341
gene_2289	multifunctional aminopeptidase A; Provisional	+	1.689	0.509	NaN	NaN	NaN	24.370	24.312	NaN	23.979	23.682	23.835
gene_3566	Flagellins polymerize to form bacterial flagella	+	1.260	-0.808	27.759	28.398	28.244	29.235	28.605	29.083	29.760	29.389	30.198
gene_167	PRK09419	+	1.720	-1.358	24.421	25.782	24.801	24.885	NaN	24.314	26.194	26.017	25.660
gene_636	thioredoxin	+	1.503	-1.411	25.298	26.021	25.093	25.853	NaN	25.292	26.674	26.832	27.444
gene_989	Superoxide dismutase	+	2.765	-2.477	28.685	27.960	27.825	26.112	25.675	NaN	28.579	28.143	28.387
gene_2147	Metal binding protein PsaA	+	2.695	-3.539	26.172	25.629	25.763	25.208	24.363	NaN	28.533	28.139	28.302
gene_1442	Inosine 5'-monophosphate dehydrogenase	+	1.924	-3.746	26.332	25.906	25.955	25.831	25.548	23.294	29.202	28.192	28.516
gene_191	Peptidase_M14_like superfamily	+	2.449	-7.015	27.666	27.811	26.726	27.128	24.128	23.485	31.990	32.249	31.547
gene_2558	GH16_lichenase		0.000	NaN	26.139	26.268	25.922	25.926	NaN	NaN	NaN	NaN	NaN

gene_2946	XynC	0.000	NaN	25.244	24.534	25.236	25.781	NaN	NaN	NaN	NaN	NaN
gene_3120	aspartate-semialdehyde dehydrogenase; Provisional.	0.000	NaN	NaN	NaN	NaN	NaN	22.908	NaN	NaN	NaN	NaN
gene_3650	The substrate-binding component of the oligopeptide-binding protein, AppA,	0.000	NaN	25.864	26.412	26.703	NaN	25.436	NaN	NaN	NaN	NaN
gene_813	Transpeptidase superfamily	0.000	NaN	NaN	NaN	NaN	27.035	NaN	NaN	NaN	NaN	NaN
gene_9	The substrate-binding component of an ABC-type dipeptide import system contains the type 2 periplasmic binding fold.	0.000	NaN	25.639	26.378	26.216	27.454	26.016	NaN	NaN	NaN	NaN
gene_1669	Polysaccharide Lyase Family 6	0.000	4.639	27.033	27.152	26.903	26.970	NaN	27.688	22.690	NaN	NaN
gene_3872	Uncharacterized proteins similar to Bacillus subtilis YncMMembers	0.692	0.753	25.657	25.218	25.902	26.943	26.063	NaN	25.952	26.054	25.245
gene_1133	Bacillus cereus group antimicrobial protein	0.000	0.551	27.427	27.256	27.722	27.090	26.996	NaN	26.492	NaN	NaN
gene_3565	Flagellins polymerize to form bacterial flagella	0.000	-0.171	25.441	25.564	25.701	NaN	NaN	27.147	27.105	26.974	27.877
gene_2779	Predicted oxidoreductase	0.000	-0.253	NaN	NaN	NaN	NaN	NaN	24.049	24.723	23.446	24.737
gene_2767	TerD	0.000	-0.513	25.667	NaN	NaN	24.830	NaN	NaN	25.641	25.240	25.148
gene_1734	Peptidases_S8_subtilisin_Vpr-like	0.403	-1.011	28.497	28.751	28.634	27.395	24.665	NaN	26.958	27.208	26.958
gene_3059	glutamine synthetase, type I.	0.000	-1.145	NaN	23.021	23.741	24.523	NaN	NaN	25.956	25.629	25.420
gene_3645	The substrate-binding component of an ABC-type oligopetide import system contains the type 2 periplasmic binding fold.	0.443	-1.606	30.336	30.131	30.594	28.844	28.412	24.020	29.126	28.561	28.407
gene_3359	NAD(P)H-nitrite reductase, large subunit	0.825	-2.007	26.563	26.684	26.885	28.187	26.233	24.397	28.555	28.549	27.735
gene_3836	Clade 1 of the heme-binding enzyme catalase	0.794	-2.482	26.629	27.029	26.907	27.741	28.190	23.695	29.126	29.322	28.626
gene_3064	N-acetylmuramoyl-L-alanine amidase	0.000	-2.770	27.018	27.062	26.794	24.868	NaN	NaN	27.909	28.053	26.953

gene_3500	Peptidases_S8_Subtilisin_subset		0.000	-4.953	25.154	24.875	24.739	24.000	NaN	NaN	29.195	28.456	29.210

Table S3 T-test results of the proteins in the secretome of *Bacillus* sp. 8A grown on bristles and hooves (BH) against proteins from *Bacillus* sp. 8A6 grown on LB medium. The LFQ ratios between conditions were formed and log2 transformed to compare the relative changes (difference). Statistical significances of abundance changes were assessed by t-test (two-tailed, heteroscedastic).

**Protein IDs**	**Function**	**Significant**	**-LOG(P-value)**	**Difference**	**LFQ intensity BH1**	**LFQ intensity BH2**	**LFQ intensity BH3**	**LFQ intensity CF1**	**LFQ intensity CF2**	**LFQ intensity CF3**	**LFQ intensity LB1**	**LFQ intensity LB2**	**LFQ intensity LB3**
gene_1796	Metallo-peptidase family M12	+	2.235	2.709	24.990	24.573	25.754	26.265	26.282	26.620	23.044	21.770	22.374
gene_3018	S1A Trypsin-like serine protease	+	2.078	2.546	27.970	27.793	28.041	27.614	27.418	28.515	25.941	25.876	24.350
gene_3746	Peptidases_S8_Subtilisin_subset	+	2.611	2.374	28.961	28.796	29.231	29.920	29.848	27.079	26.922	25.972	26.973
gene_3289	Peptidases_S8_BacillopeptidaseF-like	+	1.926	1.936	25.265	25.266	26.308	26.587	26.800	NaN	23.748	23.172	24.110
gene_1851	Flagellar biosynthesis/type III secretory pathway ATPase	+	2.247	1.878	24.499	25.120	25.367	NaN	NaN	NaN	23.100	23.528	22.725
gene_51	Rhamnogalacturan_acetylesterase_like	+	1.876	1.669	25.783	26.411	26.084	NaN	NaN	NaN	24.253	25.096	23.921
gene_3645	The substrate-binding component of an ABC-type oligopetide import system contains the type 2 periplasmic binding fold	+	2.529	1.655	30.336	30.131	30.594	28.844	28.412	24.020	29.126	28.561	28.407
gene_1734	Peptidases_S8_subtilisin_Vpr-like	+	3.852	1.586	28.497	28.751	28.634	27.395	24.665	NaN	26.958	27.208	26.958
gene_1093	M20_peptT_like	+	0.966	0.985	24.984	24.279	24.348	NaN	NaN	NaN	23.012	23.259	24.383

gene_3552	T3 Gamma-glutamyltranspeptidase	+	2.211	0.924	28.181	27.781	28.218	28.794	28.297	28.142	27.070	26.996	27.341
gene_167	PRK09419	+	1.032	-0.956	24.421	25.782	24.801	24.885	NaN	24.314	26.194	26.017	25.660
gene_2638	NADPH-dependent FMN reductase	+	1.314	-1.037	24.848	25.949	25.422	NaN	NaN	NaN	26.820	26.238	26.273
gene_636	thioredoxin	+	1.837	-1.513	25.298	26.021	25.093	25.853	NaN	25.292	26.674	26.832	27.444
gene_3359	NAD(P)H-nitrite reductase, large subunit	+	2.258	-1.569	26.563	26.684	26.885	28.187	26.233	24.397	28.555	28.549	27.735
gene_3566	Flagellins polymerize to form bacterial flagella	+	2.257	-1.649	27.759	28.398	28.244	29.235	28.605	29.083	29.760	29.389	30.198
gene_3565	Flagellins polymerize to form bacterial flagella	+	2.411	-1.750	25.441	25.564	25.701	NaN	NaN	27.147	27.105	26.974	27.877
gene_1220	Nucleoside diphosphate kinases	+	3.384	-2.130	24.166	24.688	24.222	NaN	NaN	NaN	26.288	26.650	26.528
gene_3836	Clade 1 of the heme-binding enzyme catalase	+	3.092	-2.169	26.629	27.029	26.907	27.741	28.190	23.695	29.126	29.322	28.626
gene_3059	glutamine synthetase, type I	+	2.190	-2.287	NaN	23.021	23.741	24.523	NaN	NaN	25.956	25.629	25.420
gene_2147	Metal binding protein PsaA	+	3.612	-2.470	26.172	25.629	25.763	25.208	24.363	NaN	28.533	28.139	28.302
gene_1442	Inosine 5'-monophosphate dehydrogenase	+	2.853	-2.573	26.332	25.906	25.955	25.831	25.548	23.294	29.202	28.192	28.516
gene_2293	Alanine dehydrogenase	+	3.802	-3.042	23.874	23.495	23.630	NaN	NaN	NaN	26.402	26.669	27.055
gene_3397	Peroxiredoxin (PRX) family	+	3.392	-3.201	26.491	25.976	25.678	NaN	NaN	NaN	29.096	29.596	29.055
gene_3360	branched-chain alpha-keto acid dehydrogenase	+	2.665	-3.251	NaN	23.393	22.590	NaN	NaN	NaN	26.298	26.044	26.384
gene_3500	Peptidases_S8_Subtilisin_subset	+	3.886	-4.031	25.154	24.875	24.739	24.000	NaN	NaN	29.195	28.456	29.210

gene_191	Peptidase_M14_like superfamily	+	3.472	-4.528	27.666	27.811	26.726	27.128	24.128	23.485	31.990	32.249	31.547
gene_2051	barnase	+	2.450	-4.559	22.703	24.033	NaN	NaN	NaN	NaN	27.862	28.252	27.668
gene_324	Select seq WP_098412753.1 hypothetical protein	+	3.065	-4.826	26.450	26.475	25.299	NaN	NaN	NaN	30.666	31.633	30.402
gene_1400	Glyco_hydro_11		0.000	NaN	NaN	22.399	23.576	NaN	NaN	NaN	NaN	NaN	NaN
gene_1814	acetyl-CoA acetyltransferase		0.000	NaN	NaN	24.828	NaN	NaN	NaN	NaN	NaN	NaN	NaN
gene_2212	3-hydroxyacyl-CoA dehydrogenase		0.000	NaN	NaN	23.061	23.382	NaN	NaN	NaN	NaN	NaN	NaN
gene_2558	GH16_lichenase		0.000	NaN	26.139	26.268	25.922	25.926	NaN	NaN	NaN	NaN	NaN
gene_2838	ETHE1 (PDO type I), persulfide dioxygenase A		0.000	NaN	NaN	NaN	23.148	NaN	NaN	NaN	NaN	NaN	NaN
gene_2878	Arginase		0.000	NaN	20.629	NaN	22.202	NaN	NaN	NaN	NaN	NaN	NaN
gene_2883	ranspeptidase superfamily		0.000	NaN	NaN	20.862	NaN	NaN	NaN	NaN	NaN	NaN	NaN
gene_2906	Both mitochondrial acyl-CoA dehydrogenases (ACAD) and peroxisomal acyl-CoA oxidases		0.000	NaN	NaN	NaN	22.365	NaN	NaN	NaN	NaN	NaN	NaN
gene_2945	GH43_AXH_1		0.000	NaN	24.973	25.057	24.582	NaN	NaN	NaN	NaN	NaN	NaN
gene_2946	XynC		0.000	NaN	25.244	24.534	25.236	25.781	NaN	NaN	NaN	NaN	NaN
gene_3081	Peptidases_S8_1		0.000	NaN	NaN	26.497	27.039	NaN	NaN	NaN	NaN	NaN	NaN
gene_3376	Peptidase_M29		0.000	NaN	NaN	23.520	NaN	NaN	NaN	NaN	NaN	NaN	NaN
gene_3650	The substrate-binding component of the oligopeptide-binding protein, AppA,		0.000	NaN	25.864	26.412	26.703	NaN	25.436	NaN	NaN	NaN	NaN
gene_635	Alpha-L-arabinofuranosidase		0.000	NaN	26.334	26.536	26.432	NaN	NaN	NaN	NaN	NaN	NaN
gene_838	hypothetical protein		0.000	NaN	NaN	NaN	25.538	NaN	NaN	NaN	NaN	NaN	NaN
gene_9	The substrate-binding component of an ABC-type dipeptide import system contains the type 2 periplasmic binding fold		0.000	NaN	25.639	26.378	26.216	27.454	26.016	NaN	NaN	NaN	NaN

gene_992	Substrate binding domain of putative ABC-type phosphate transporter	0.000	NaN	NaN	25.722	24.913	NaN	NaN	NaN	NaN	NaN	NaN
gene_1669	Polysaccharide Lyase Family 6 is a family of beta-helical polysaccharide lyases	0.000	4.339	27.033	27.152	26.903	26.970	NaN	27.688	22.690	NaN	NaN
gene_525	Triacylglycerol esterase/lipase EstA	0.848	1.188	24.793	24.135	23.738	NaN	NaN	NaN	NaN	22.431	23.637
gene_1133	Bacillus cereus group antimicrobial protein	0.000	0.977	27.427	27.256	27.722	27.090	26.996	NaN	26.492	NaN	NaN
gene_1853	F0F1 ATP synthase subunit beta; Validated	0.720	0.748	24.951	25.338	25.916	NaN	NaN	NaN	25.031	25.044	23.885
gene_642	succinate dehydrogenase flavoprotein subunit;	0.646	0.614	24.637	24.426	24.367	NaN	NaN	NaN	24.454	24.088	23.046
gene_334	co-chaperone GroES (Cpn10) binds to Cpn60 in the presence of Mg-ATP and suppresses the ATPase activity of the latter	0.491	0.517	NaN	24.596	24.369	NaN	NaN	NaN	23.636	23.630	24.632
gene_1629	peptidase T	0.781	0.496	24.354	NaN	24.074	NaN	NaN	NaN	23.976	23.337	23.839
gene_3118	dihydrodipicolinate synthase	0.641	0.432	23.943	23.923	23.995	NaN	NaN	NaN	23.182	23.255	24.126
gene_2767	TerD	0.000	0.324	25.667	NaN	NaN	24.830	NaN	NaN	25.641	25.240	25.148
gene_2107	enolase	0.820	0.257	26.171	26.625	26.268	NaN	NaN	NaN	26.091	26.024	26.179
gene_1043	Xaa-Pro aminopeptidase	0.000	-0.100	NaN	23.364	NaN	NaN	NaN	NaN	23.497	23.729	23.167
gene_3872	Uncharacterized proteins similar to Bacillus subtilis Ync	0.186	-0.158	25.657	25.218	25.902	26.943	26.063	NaN	25.952	26.054	25.245
gene_989	Superoxide dismutase	0.292	-0.213	28.685	27.960	27.825	26.112	25.675	NaN	28.579	28.143	28.387
gene_643	succinate dehydrogenase iron-sulfur subunit	0.368	-0.223	23.482	23.574	23.224	NaN	NaN	NaN	24.111	23.413	23.425
gene_3436	phosphocarrier protein HPr	0.000	-0.486	NaN	21.275	NaN	NaN	NaN	NaN	NaN	22.620	20.903
gene_1774	phosphotransacetylase	0.584	-0.554	NaN	24.059	23.372	NaN	NaN	NaN	23.884	24.707	24.218
gene_3064	N-acetylmuramoyl-L-alanine amidase or MurNAc-LAA	0.893	-0.680	27.018	27.062	26.794	24.868	NaN	NaN	27.909	28.053	26.953
gene_1887	(3R)-hydroxymyristoyl-ACP dehydratase	0.000	-0.779	22.718	NaN	NaN	NaN	NaN	NaN	23.330	23.982	23.180

gene_570	mainly hydrolytic enzymes and related proteins	0.000	-1.169	NaN	24.357	NaN	NaN	NaN	NaN	25.518	25.899	25.161
gene_1076	Glutamate dehydrogenase/leucine dehydrogenase	0.000	-1.348	24.316	NaN	NaN	NaN	NaN	NaN	25.998	25.724	25.268
gene_1773	putative heme peroxidase	0.000	-2.303	NaN	22.577	NaN	NaN	NaN	NaN	24.312	25.469	24.860
gene_2352	glucose-6-phosphate isomerase	0.000	-2.920	22.525	NaN	NaN	NaN	NaN	NaN	24.749	25.890	25.696

Table S4 The accession numbers of the Hotpep predicted protease nucleotide sequences in *Bacillus* sp. 8A6 genome and the up-regulated protein nucleotide sequences in the secretome when *Bacillus* sp. 8A6 was grown in keratin media compared to LB medium

**Gene ID**	**Accession number**
gene_3268_A08_2	MH475952
gene_933_A25_1	MH475953
gene_3288_A36_5	MH475954
gene_314_C26_1	MH475955
gene_404_C26_2	MH475956
gene_3262_C26_3	MH475957
gene_3666_C26_3	MH475958
gene_1820_C26_4	MH475959
gene_1438_C26_6	MH475960
gene_3665_M20	MH475961
gene_2038_C26_40	MH475962
gene_1227_S10	MH475963
gene_403_C26_79	MH475964
gene_12_C39_1	MH475965
gene_1808_C39_1	MH475966
gene_1408_C39_1	MH475967
gene_3803_C39_1	MH475968
gene_3642_C39_1	MH475969
gene_3652_C39_1	MH475970
gene_3804_C39_2	MH475971
gene_1087_C39_2	MH475972
gene_2680_C39_2	MH475973
gene_1697_C39_2	MH475974
gene_2221_C39_2	MH475975
gene_1409_C39_2	MH475976
gene_443_C39_2	MH475977
gene_2117_C39_2	MH475978
gene_547_C39_3	MH475979
gene_3548_C39_3	MH475980
gene_10_C39_6	MH475981
gene_3651_C39_6	MH475982
gene_3522_C39_6	MH475983
gene_3799_C39_6	MH475984
gene_3641_C39_6	MH475985
gene_2207_C39_6	MH475986
gene_3887_C39_6	MH475987
gene_2702_C39_6	MH475988
gene_2168_C39_6	MH475989
gene_2092_C39_9	MH475990
gene_1690_C39_9	MH475991
gene_3733_C39_9	MH475992
gene_1875_C39_12	MH475993
gene_465_C39_12	MH475994

gene_2669_C39_12	MH475995
gene_1613_C39_12	MH475996
gene_3384_C39_12	MH475997
gene_1515_C39_12	MH475998
gene_2015_C39_12	MH475999
gene_2066_C39_12	MH476000
gene_1667_C39_12	MH476001
gene_2502_C39_12	MH476002
gene_821_C39_12	MH476003
gene_1660_C39_12	MH476004
gene_2065_C39_18	MH476005
gene_3881_C39_20	MH476006
gene_1359_C40_18	MH476007
gene_3824_C40_18	MH476008
gene_1917_C40_34	MH476009
gene_3523_C40_48	MH476010
gene_2043_C40_112	MH476011
gene_283_C44_1	MH476012
gene_2872_C44_2	MH476013
gene_1411_C44_3	MH476014
gene_453_C44_4	MH476015
gene_3703_C44_6	MH476016
gene_1938_S26	MH476017
gene_285_C26	MH476018
gene_166_C56_7	MH476019
gene_2367_C56_11	MH476020
gene_219_C56_37	MH476021
gene_3856_C60_16	MH476022
gene_2682_C60_16	MH476023
gene_1661_C60_28	MH476024
gene_3414_C82_19	MH476025
gene_1089_C82_24	MH476026
gene_1454_U62	MH476027
gene_3633_M03_2	MH476028
gene_1991_M03_4	MH476029
gene_1605_M06_1	MH476030
gene_1008_M14_10	MH476031
gene_191_M14_40	MH476032
gene_1342_M15_2	MH476033
gene_903_M15_15	MH476034
gene_76_M15_36	MH476035
gene_3109_M16_8	MH476036
gene_3125_M16_10	MH476037
gene_3110_M16_53	MH476038
gene_2289_M17_1	MH476039
gene_1629_M20_2	MH476040
gene_1093_M20_7	MH476041
gene_3400_M20_9	MH476042

gene_2668_M20_11	MH476043
gene_2753_M20_21	MH476044
gene_493_M20_23	MH476045
gene_272_M20_56	MH476046
gene_3764_M20_66	MH476047
gene_1112_M20_69	MH476048
gene_343_M42	MH476049
gene_1839_S12	MH476050
gene_185_A22	MH476051
gene_345_M16	MH476052
gene_2260_M23_46	MH476053
gene_720_M23_74	MH476054
gene_1872_M23_91	MH476055
gene_706_M23_94	MH476056
gene_3629_M23_110	MH476057
gene_1375_M23_129	MH476058
gene_1997_M23_148	MH476059
gene_1043_M24_3	MH476060
gene_1989_M16	MH476061
gene_181_M24_12	MH476062
gene_3441_M24_60	MH476063
gene_2494_M24_75	MH476064
gene_3376_M29_1	MH476065
gene_1278_M32_1	MH476066
gene_3263_M38_2	MH476067
gene_2024_M38_18	MH476068
gene_3261_I39	MH476069
gene_270_M38_32	MH476070
gene_3369_M38_45	MH476071
gene_398_M41_1	MH476072
gene_617_M42_1	MH476073
gene_506_M42_1	MH476074
gene_3754_M42_2	MH476075
gene_3480_M48_2	MH476076
gene_3157_M50_4	MH476077
gene_707_M50_7	MH476078
gene_1798_M50_26	MH476079
gene_3530_M55_2	MH476080
gene_3153_M13	MH476081
gene_802_S12	MH476082
gene_1027_M73_2	MH476083
gene_335_M79_15	MH476084
gene_1197_M82_1	MH476085
gene_1796_M12	MH476086
gene_3079_M86_1	MH476087
gene_3083_M86_1	MH476088
gene_3560_M86_1	MH476089
gene_3175_N06_4	MH476090

gene_1498_N10_1	MH476091
gene_852_N10_1	MH476092
gene_577_N10_2	MH476093
gene_1444_N10_5	MH476094
gene_2986_N10_5	MH476095
gene_3133_N10_11	MH476096
gene_1445_N10_13	MH476097
gene_2987_N10_13	MH476098
gene_2464_N10_16	MH476099
gene_3202_N10_18	MH476100
gene_3532_S01_2	MH476101
gene_1509_S01_2	MH476102
gene_2204_S01_110	MH476103
gene_3018_S01_202	MH476104
gene_3746_S08_1	MH476105
gene_1139_S08_1	MH476106
gene_3081_S08_6	MH476107
gene_3500_S08_20	MH476108
gene_1734_S08_30	MH476109
gene_2811_S08_38	MH476110
gene_2799_S08_38	MH476111
gene_3289_S08_48	MH476112
gene_1594_S08_66	MH476113
gene_2555_S09_6	MH476114
gene_2722_S09_36	MH476115
gene_449_S09_50	MH476116
gene_1473_S09_85	MH476117
gene_2272_S09_93	MH476118
gene_1472_I39	MH476119
gene_1131_S09_174	MH476120
gene_2677_S09_198	MH476121
gene_2127_S09_202	MH476122
gene_1173_S11_4	MH476123
gene_1146_S11_6	MH476124
gene_1441_S11_8	MH476125
gene_2883_S12_7	MH476126
gene_2884_S16	MH476127
gene_2501_T02	MH476128
gene_813_S12_90	MH476129
gene_2698_A01	MH476130
gene_1489_S33	MH476131
gene_2699_S33	MH476132
gene_2696_S26	MH476133
gene_2697_S33	MH476134
gene_1420_S13_9	MH476135
gene_2058_S14_1	MH476136
gene_3116_S14_9	MH476137
gene_1567_M20	MH476138

gene_2862_C44	MH476139
gene_2868_S09	MH476140
gene_2597_I63	MH476141
gene_2109_M42	MH476142
gene_3028_T02	MH476143
gene_682_S16_1	MH476144
gene_2443_S16_2	MH476145
gene_681_S16_5	MH476146
gene_2442_S16_5	MH476147
gene_3315_S16_6	MH476148
gene_342_T03	MH476149
gene_3012_S24_2	MH476150
gene_3890_S26_38	MH476151
gene_3728_S26_41	MH476152
gene_3379_S26_48	MH476153
gene_1026_S26_59	MH476154
gene_1485_S09	MH476155
gene_456_S33_70	MH476156
gene_1494_S09	MH476157
gene_2470_S33_142	MH476158
gene_2755_S33_169	MH476159
gene_2694_S33_192	MH476160
gene_2790_S33_193	MH476161
gene_2695_I43	MH476162
gene_2232_S33_242	MH476163
gene_2345_S33_264	MH476164
gene_420_S33_287	MH476165
gene_1999_S41_4	MH476166
gene_1345_S41_9	MH476167
gene_558_S49_7	MH476168
gene_948_S49_24	MH476169
gene_494_S51_3	MH476170
gene_1004_S54_10	MH476171
gene_2585_S54_13	MH476172
gene_1067_S55_1	MH476173
gene_3525_S66_3	MH476174
gene_1809_S66_7	MH476175
gene_1806_S66_9	MH476176
gene_2520_S66_23	MH476177
gene_2521_S66_23	MH476178
gene_3199_T01_16	MH476179
gene_3552_T03_1	MH476180
gene_3669_T05_1	MH476181
gene_774_U32_1	MH476182
gene_773_U32_7	MH476183
gene_370_U57_1	MH476184
gene_3645	MH476185
gene_3650	MH476186

gene_9	MH476187
gene_992	MH476188
